# Promising antibiofilm formation: Liquid phase pulsed laser ablation synthesis of Graphene Oxide@Platinum core-shell nanoparticles

**DOI:** 10.1371/journal.pone.0310997

**Published:** 2024-09-24

**Authors:** Buthenia A. Hasoon, Dahlia M. A. Hasan, Kareem H. Jawad, Saaud S. Shakaer, Ghassan M. Sulaiman, Nehia N. Hussein, Hamdoon A. Mohammed, Mosleh M. Abomughaid, Thotakura Ramesh

**Affiliations:** 1 Department of Applied Sciences, University of Technology, Baghdad, Iraq; 2 Department of Biomedical Engineering, Technology University, Baghdad, Iraq; 3 Department of Laser and Optoelectronics Engineering, University of Technology, Baghdad, Iraq; 4 Department of Medicinal Chemistry and Pharmacognosy, College of Pharmacy, Qassim University, Buraydah, Qassim, Saudi Arabia; 5 Department of Medical Laboratory Sciences, College of Applied Medical Sciences, University of Bisha, Bisha, Saudi Arabia; 6 Department of Physics, BVRIT Hyderabad College Engineering for Women, Hyderabad, India; Fraunhofer USA, Inc. Center Midwest, UNITED STATES OF AMERICA

## Abstract

The increasing prevalence of multi-drug resistance in pathogenic bacteria has rendered antibiotics ineffective, necessitating the exploration of alternative antibacterial approaches. Consequently, research efforts have shifted towards developing new antibiotics and improving the efficacy of existing ones. In the present study, novel core shell graphene oxide@platinum nanoparticles (GRO@Pt-NPs) and their unchanging form have been synthesized using the two-step pulsed laser ablation in liquid (PLAL) technique. The first step involved using the graphene target to create graphene nanoparticles (GRO-NPs), followed by the ablation of GRO-NPs inside platinum nanoparticles (Pt-NPs). To characterize the nanoparticles, various methods were employed, including UV-VIS, transmission electron microscopy (TEM), energy dispersive X-ray (EDX), mapping tests, and X-ray diffraction (XRD). The anti-bacterial and anti-biofilm properties of the nanoparticles were investigated. TEM data confirm the creation of GRO@Pt-NPs. The average particle size was 11 nm for GRO-NPs, 14 nm for Pt-NPs, and 26 nm for GRO@Pt-NPs. The results demonstrate that the created GRO@Pt-NPs have strong antibacterial properties. This pattern is mostly produced through the accumulation of GRO@Pt-NPs on the bacterial surface of *Klebsiella pneumoniae* (*K*. *pneumoniae*) and *Enterococcus faecium* (*E*. *faecium*). The inhibition zones against *K*. *pneumoniae* and *E*. *faecium* when GRO-NPs were used alone were found to be 11.80 mm and 11.50 mm, respectively. For Pt-NPs, the inhibition zones of *E*. *faecium* and *K*. *pneumoniae* were 20.50 mm and 16.50 mm, respectively. The utilization of GRO@Pt-NPs resulted in a significant increase in these values, with inhibitory rates of 25.50 mm for *E*. *faecium* and 20.45 mm for *K*. *pneumoniae*. The antibacterial results were more potent in the core–shell structure than the GRO-NPs alone or Pt-NPs alone. The current work uses, for the first time, a fast and effective technique to synthesize the GRO@Pt-NPs by PLAL method, and the preparation has high clinical potential for prospective use as an antibacterial agent.

## 1. Introduction

Nanomaterial synthesis plays a crucial role in the playing field of nanotechnology, with the synthesis methods focusing on exploring new physical possessions and applications of nanomaterials and nanostructures. The primary objective of these synthesis methods is to achieve specific sizes, shapes, crystal microstructures, and chemical compositions [1]. One of these methods is the production of nanoparticles, also known as NPs, in a gaseous or liquid environment using laser ablation in a solution (Pulsed Laser Ablation in Liquid Media (PLAL)) of a solid target and the resulted NPs are gathered in colloidal or nanoscale forms. When contrasted to other methods, this technique in NP synthesis/generation stands out for its ease of use, rapidity, and directness. The technique also enables the production of the nanoparticles in a single step without the need for chemical precursors. In addition, PLAL is a potential technique for better control over nanoparticle size, shape, and composition. Beside previous advantages, the technique provides a possibility of in-situ surface functionalization during synthesis and an environmentally friendly "green" synthesis approach. Importantly, this removes the need for prolonged response times, high temperatures, or complex multi-step biochemical mixtures [2]. The nanomaterials utilized can be generally useful for a diversity of uses, including medical analysis and image processing, sensors, electrical parts, catalysts, and even medications and antibacterial properties, due to their characteristic chemical, physical, and optoelectronic possessions [3]. Platinum nanoparticles (Pt-NPs) find applications in the biological, chemical, therapeutic, and electronic sectors due to their remarkable constancy, excellent biological compatibility, and surface interaction [4]. Platinum is a systematically studied class of metal that has attracted considerable interest from scientists across many industries due to the unique catalytic characteristics of the nanoparticles formed from it. Applications of Pt-NPs are generally influenced by factors such as size, shape, and dispersion. These nanoparticles catalyze a number of chemical and organic procedures, like the oxidation and hydrolysis processes. Furthermore, they function as electrocatalysts in processes involving the reduction of dioxygen [5]. Smaller nanoparticles, particularly Pt-NPs produced in sizes smaller than 5 nm, are highly effective in killing bacteria due to their electro-catalytic properties. They generate reactive oxygen species that cause bacterial cell death. It is important to note that antibiotic resistance is rapidly spreading among bacteria through horizontal gene transfer [6,7]. Besides the potential killing activity of graphene-based materials on a wide variety of germs, they have several benefits. They are being simple to prepare, renewable, and having special catalytic qualities. They also have exceptional physical qualities like mechanical strength, a large specific surface area, optical clarity, and thermal and electrical conductivity [8–11]. Graphene oxide (GRO-NPs) can be combined with metal nanoparticles to produce nanocomposites with greater active surface regions [12–14]. This increases electron transport and catalyzing methods, which can be applied to fuel cells, sensors, optical devices, and electronics [14,15]. The combined antibacterial properties of GRO-NPs and Pt-NPs in their combined form are higher than the sum of each of them. Graphene oxide’s huge surface area and outstanding electrical conductivity enhance the creation of reactive oxygen species (ROS) and the efficiency of bacterial inactivation when paired with the catalytic properties of platinum nanoparticles. These synergistic activities result in an improved antimicrobial effect for the complex [16,17]. But there are still a lot of unrequited concerns about the mechanism of action, the importance of size and composition in relation to bacterial activity, toxicity guidelines, and other pertinent topics. GRO-NPs and Pt-NPs have confirmed remarkable effectiveness as anti-bacterial agents against a diverse array of microorganisms. In that context, some types of microbes have been tested, for example, *S*. *aurous* and *P*. *aerogenosa*. This activity’s broad spectrum indicates that these composites may be useful in treating bacterial infections and preventing the spread of strains of bacteria that are resistant to antibiotics [18,19].

NPs have proven to be effective in the biomedical field, specifically in the development of nanomedicines for treating bacterial infections like *S*. *aurous* [20–22]. Their effectiveness lies in their capacity to stop biofilm creation, penetrate cell and biofilm membranes, increase intracellular retention, and enhance the action of antibacterial agents that are loaded onto them [23]. In addition, NPs drug delivery systems offer a promising solution for addressing the difficulties associated with treating bacterial infections, including the antibiotic resistance[24,25]. Platinum is used due to its desirable physical and chemical properties, such as high melting point, corrosion resistance, catalytic activity, and thermal stability, which made it suitable for their nanoparticle synthesis and characterization experiments [4,5]. To the best of our understanding, no earlier studies have created the GRO@Pt-NPs using the technique described in this work, which employs a fast and effective technique known as pulsed laser ablation in liquid (PLAL). The main objective of this study is to propose a new hybrid method for producing the GRO@Pt-NPs by a core-shell structure and Nd:YAG laser. The synthesis includes two steps: first, laser ablation of the graphene target to produce GRO-NPs (core) colloidal, then another step of Pt (shell) ablation within the GRO-NPs colloidal and studying its biological applications.

## 2. Methodology

### 2.1. Materials

Graphene (GR) and Platinum (Pt) plates were obtained from Sigma-Aldrich with 99.98% purity, dimensions 1 × 1 cm^2^.

### 2.2. Synthesis of GRO-NPs

The graphene target was placed in a glass beaker containing deionized distilled water (D.D.W). Laser ablation of the graphene target was performed using the PLAL technique. The PLAL process was carried out for 10 minutes at a temperature of 25°C. An Nd:YAG laser operating at a wavelength of 1064 nm was used for the laser ablation. The laser parameters were set as follows: Laser energy: 500 mJ. Repetition rate: 7 Hz. Pulse duration 10 ns. A lens with a focal length of 9 cm and a spot size of 2.5 mm was used to focus the laser beam onto the graphene target. This process resulted in the formation of graphene oxide nanoparticles (GRO-NPs) in the D.D.W. solution.

### 2.3. Synthesis of GRO@Pt-NPs

The platinum (Pt) plate was then placed in the D.D.W. solution containing the GRO-NPs. The Pt target was exposed to the same laser ablation conditions as used for the GRO-NPs synthesis. The PLAL process was carried out for the Pt target in the presence of the GRO-NPs. This resulted in the formation of the GRO@Pt-NPs. The GRO@Pt-NPs solution was centrifuged at 5000 g for10 min, and was washed excessively with D.D.W, to remove any impurities. Finally, it was placed in an oven at 60°C, for 12 h. [26].

### 2.4. Characterization of GRO@Pt-NPs

The UV-vis spectrum was measured using an SP8001 spectrophotometer (Japan) within the range of 100–600 nm to determine the optical properties, including absorbance. The samples were prepared in a standard quartz cuvette, and the spectra were recorded at room temperature. A baseline correction was applied using a reference solvent [21]. For crystalline samples, XRD analysis was conducted using a Lab XRD-6100 (Shimadzu Corporation, China) with Cu Kα radiation (wavelength 0.154 nm). The diffraction patterns were recorded within the angle of 10° to 70° [26]. Atomic absorption spectroscopy (AAS) was used to measure the concentration of metal elements. The AAS was done at the College of Science, Physical Department, University of Baghdad, Iraq. The AAS measurements were taken using a standard calibration curve specifically recognized for each element being investigated [27]. TEM (Hitachi microscope, Tokyo, Japan) analysis was used to study the crystal structure and shape of the nanoparticles at the nanoscale [27]. EDX and mapping were used to investigate the elemental composition. A JSM-IT800 device was used for EDX assessment [26].

### 2.5. Anti-bacterial activity assay

*K*. *pneumoniae* and *E*. *faecium* were used for antibacterial experiments to assess the potential of GRO@Pt-NPs as alternative antimicrobial agents, revealing their broad-spectrum antibacterial activities. Pt-NPs, GRO-NPs, and GRO@Pt-NPs were tested for their antibacterial efficacy against *K*. *pneumoniae* and *E*. *faecium*. First, the bacterial strains were cultured on Mueller-Hinton agar medium and kept for 24 hours at 37°C. Three wells, each about 6 mm in diameter, were made in the agar using micropipette tips. The corresponding nanoparticle suspensions of 33.30 ppm for Pt-NPs and 25.3 ppm for GRO-NPs were then added to each of these wells. The diameter of the inhibition zones was used to gauge how well NPs prevented bacterial growth after the plates were stored for 24 hours. D.D.W. was used as the negative control [27,28].

### 2.6. Biofilm test

The GRO-NPs, Pt-NPs, and GRO@Pt-NPs were applied to bacterial strains that had been cultivated in 96-well plates at concentrations of 1xI0 ^6^ and incubated for a full day. The samples underwent cleaning. The adhering bacteria were stained with crystal violet (C.V.) at a concentration of 0.1%, and then they were twice washed with DW. To determine the growth of biofilm, 0.2 ml of 95% ethanol was added to the C.V.-stained wells. The wells were subsequently shaken for a period of two hours. The optical density was calculated at 595 nm [29,30].

### 2.7. Statistical analysis

The obtained data were statistically analyzed by GraphPad Prism 6. The data values were calculated as the mean ± SD of three experiments. Differences were set at * p ≤ 0.05, ** p ≤ 0.01, or *** p ≤ 0.001.

## 3. Result and discussion

### 3.1. Synthesis and investigation of NPs’ morphology

**Fig 1:**
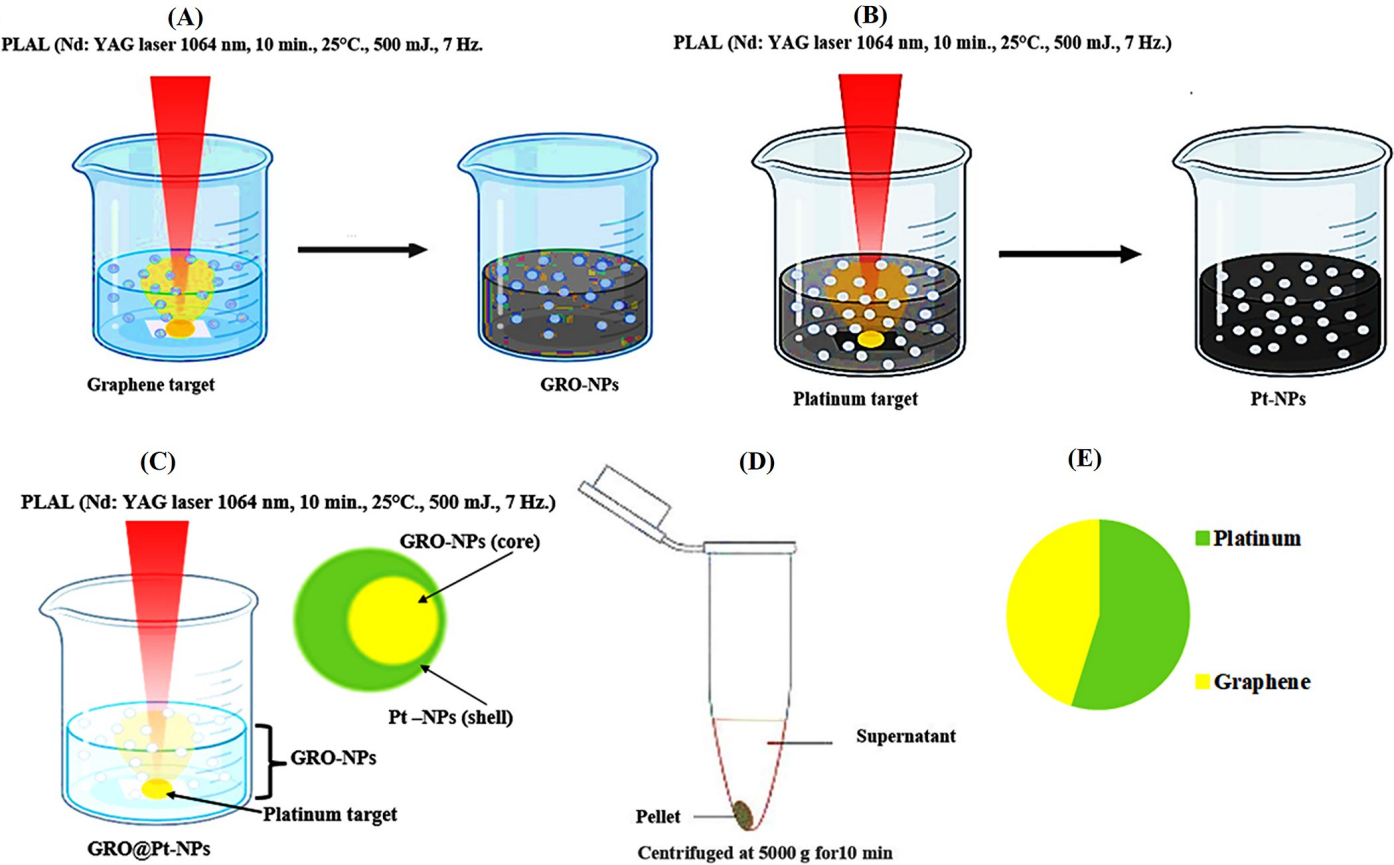
Diagram describes the production of GRO-NPs@Pt-NPs by the PLAL technique. (A) GRO-NPs; (B) Pt-NPs, (C) GRO@Pt-NPs, (D) purification by centrifugation, (E) the ratio% of GRO@Pt-NPs.

Core-shell GRO@Pt-NPs were effectively produced by the PLAL technique and exhibited a noticeable color shift (Fig 1A–1E). When the GRO-NPs were originally synthesized in D.D.W., the color of the water changed to a light brown, and when the core-shell of GRO@Pt-NPs formed, the water’s color became darker. The NP concentration was determined by atomic absorption spectroscopy (AAS). The method of AAS is widely used in analytical chemistry to determine the presence of trace metals in a variety of sample types, including environmental, biological, and industrial samples. AAS is a particular device that consists of a radiation source, atomizer, monochromatic, and sensor. There are numerous types of sprays that can be used in AAS, including flame, electrocurrent, and vapor generation sprays [31]. Out of the total concentration, 56.8% was made up of Pt concentration (33.30 ppm) and 43.2% was made up of GRO concentration (25.3 ppm). This percentage shows that Pt particles were present on GRO. The two stages that were taken in this work to create core-shell NPs are shown in Fig 1. Prior to the production of the GRO liquid, which interacts with the evaporated water molecules to make GRO-NPs, the GR target was ablated with the laser pulse. This produced a high temperature on the GR plate because of the laser pulse’s condensation. Furthermore, the process of current evaporation and the creation of Pt-encapsulated GRO@Pt-NPs are critical to the ablation of Pt in GR colloids. 1B and 1C demonstrate how the GRO@Pt-NPs are created. Ablated platinum ions, and ionized GRO-NPs from Pt-NPs interact to form a rounded-like structure on the surface of the GRO-NPs. Pt-NPs shells and GRO@Pt-NPs cores were produced as a result of this process [32].

### 3.2. Characterization of nanoparticles

#### 3.2.1. UV-Vis spectroscopy assay

**Fig 2:**
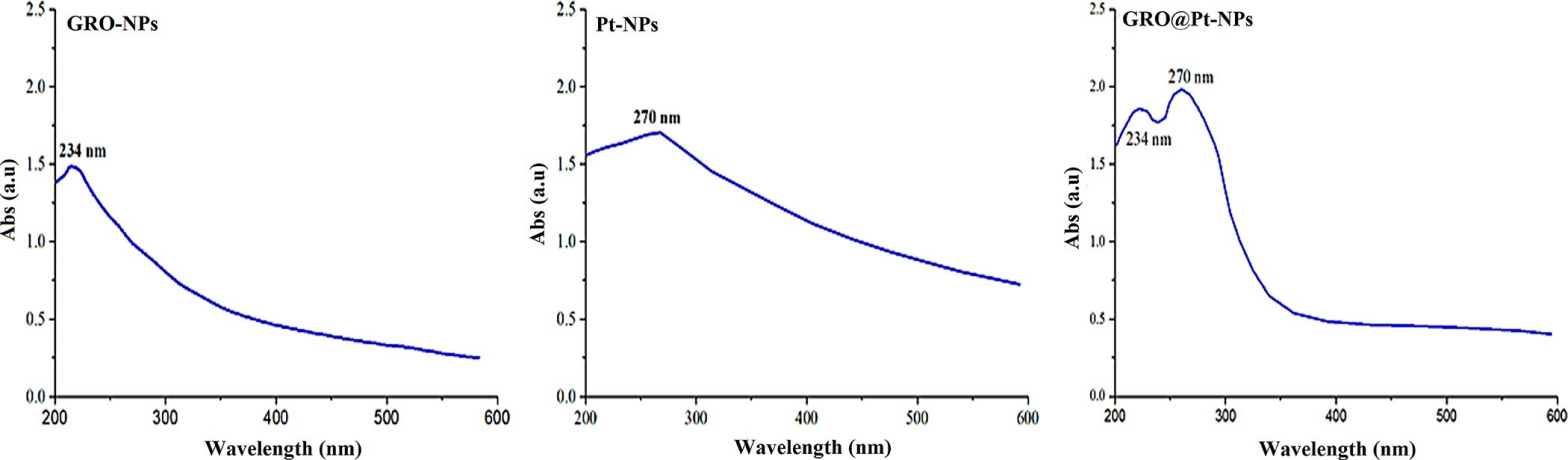
UV-vis spectrum analysis of GRO-NPs, Pt-NPs, and GRO@Pt-NPs.

The UV-Vis spectra of GRO-NPs, Pt-NPs, and GRO@Pt-NPs were recorded and are presented in Fig 2. The spectra reveal distinct absorption peaks corresponding to each type of nanoparticle, indicating successful synthesis and unique optical properties. For GRO-NPs, an absorption peak was observed at 234 nm. This peak is characteristic of the graphene oxide structure, indicating the presence of conjugated π-electron systems within the nanoparticles. Pt-NPs displayed an absorption peak at 270 nm, which is typical for platinum nanoparticles. This peak rises from the surface plasmon resonance (SPR) of platinum, confirming the creation of Pt-NPs at a small particle size. The GRO@Pt-NPs exhibited two absorption peaks at 234 and 278 nm. The GRO-NPs component of the nanoparticles was recognized as the peak at 234 nm in their UV-visible. A peak at 278 nm was noted, which, in comparison to the absorption spectrum of pure Pt-NPs, indicated a minor red shift. The communication between the Pt-NPs and GRO is responsible for this shift in the absorption peak, representing that the two materials were successfully combined and that this may have different electrical characterizations. Furthermore, a blue shift in these nanoparticles’ absorption edges was noticed, signifying the creation of smaller-sized nanoparticles. This blue shift is a typical occurrence in nanoscale materials due to quantum confinement properties produced by smaller particles, which alter the optical characteristics. The spectra’s distinct absorption peaks and their shifting patterns verify the combination of nanoparticles with certain sizes and connections, which are essential for the particles’ possible uses in a variety of disciplines [33].

#### 3.2.2. Transmission electron microscope assay

**Fig 3:**
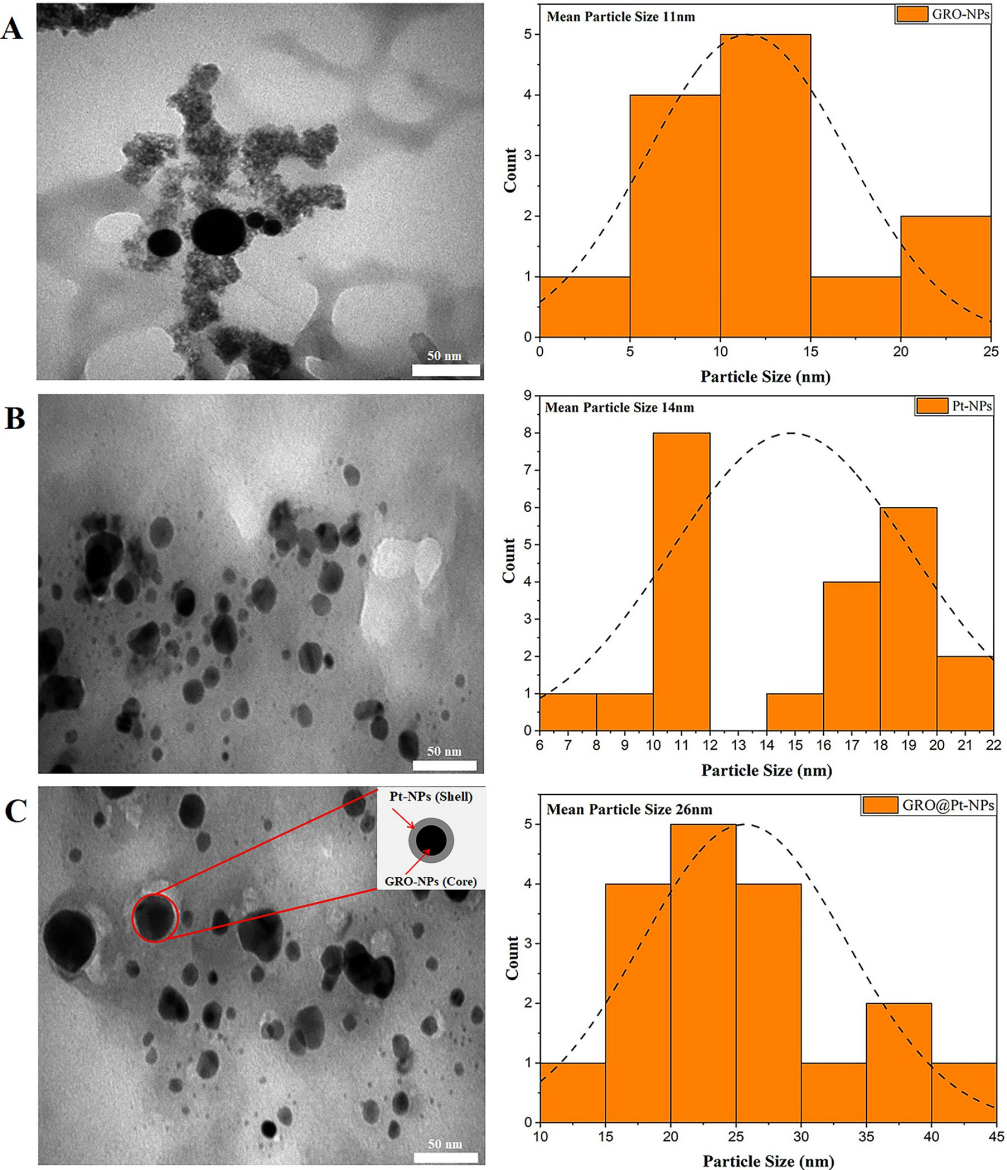
TEM images and particle size distribution histograms for (A) GRO-NPs, (B) Pt-NPs, and (C) GRO@Pt-NPs.

The TEM image of GRO-NPs with an average diameter of 11 nm and a diameter reaching from 4 to 22 nm was produced, with spherical-shaped nanoparticles predominating (Fig 3A). The size distribution of Pt-NPs with a diameter ranging from 5 to 30 nm (an average of 14 nm) is shown in Fig 3B, indicating the successful creation of sphere-shaped nanoparticles. GRO@Pt-NPs with a size distribution ranging from 10 to 45 nm (an average of 26 nm) were obtained, and NPs in the shape of spheres were successfully overcome (Fig 3C). To calculate the average particle size, we used 2–3 samples having around ~50 particles and image J software (Java 1.8.0, Gaithersburg, MD, USA) was conducted to determine the particle diameter. As illustrated in Fig 3, PLAL utilizing millisecond lasers typically leads to an increase in medium temperatures, whereas ablation is carried out with shorter pulses (such as nanosecond and femtosecond lasers) than the thermal evaporation mechanism for graphene (GR), resulting in the creation of sphere-shaped nanoparticles (NPs) [33,34]. Furthermore, the ablation of pyrolytic platinum (Pt.) in graphene oxide (GRO) colloids relies on the process of thermal evaporation and the generation of Pt-encapsulated GRO-NPs. As depicted in Fig 3C, the formation of GRO@Pt-NPs occurs.

The interaction between ablated graphene ions, water vapor, and ionized graphene nanoparticles (GRO-NPs) derived from graphene colloids leads to the creation of a spherical-like structure on the surface of the GRO-NPs. This process ultimately results in the formation of shells composed of pyrolytic graphite nanoparticles and cores composed of GRO-NPs encapsulated by Pt-NPs (as shown in Fig 3C). The mechanism of core-shell creation observed in this study resembles the findings reported, who investigated the creation of core-shell nanoparticles consisting of cores and shells [35]. Graphene nanoparticles (GRO-NPs), as depicted in Fig 3B, have a round form and significant accumulation as a result of interparticle relationships, which is in accordance with TEM meanings. Because of the nanoparticles’ high surface-to-volume percentage, attraction physical forces drive this relationship [36]. On the other hand, Fig 3C portrays the morphology of graphene-encapsulated platinum nanoparticles (GRO-@Pt-NPs), which feature a near-smooth surface and minimal accumulation. This is attributed to the protective layer of graphene that covers the surface of the nanoparticles, preventing significant interparticle interactions and subsequent agglomeration.

#### 3.2.3. EDX investigation

**Fig 4:**
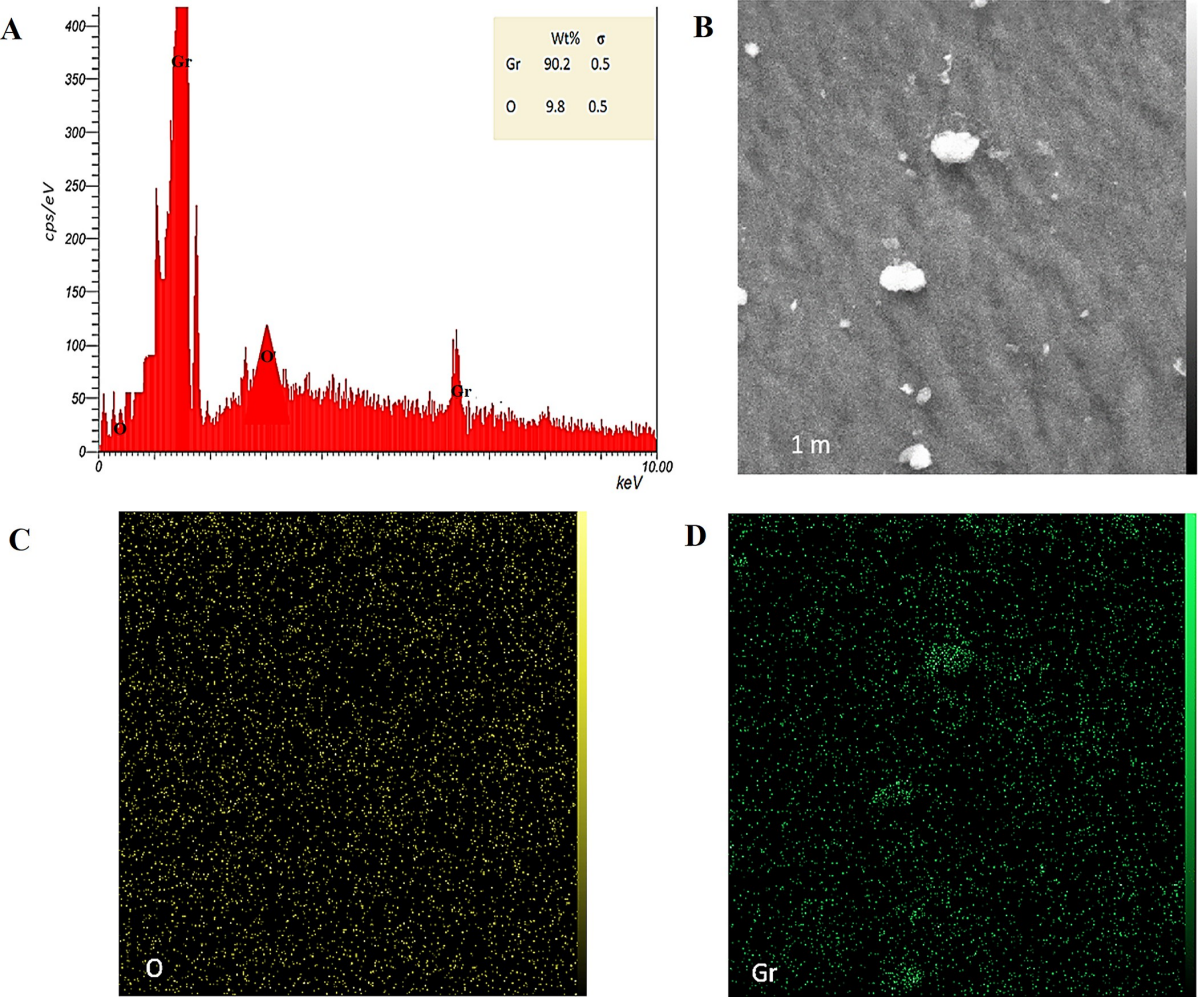
(A) EDX analysis for GRO-NPs showing EDX spot (B) and elemental mapping (C, D) of oxygen (O) and graphene (Gr), respectively.

**Fig 5:**
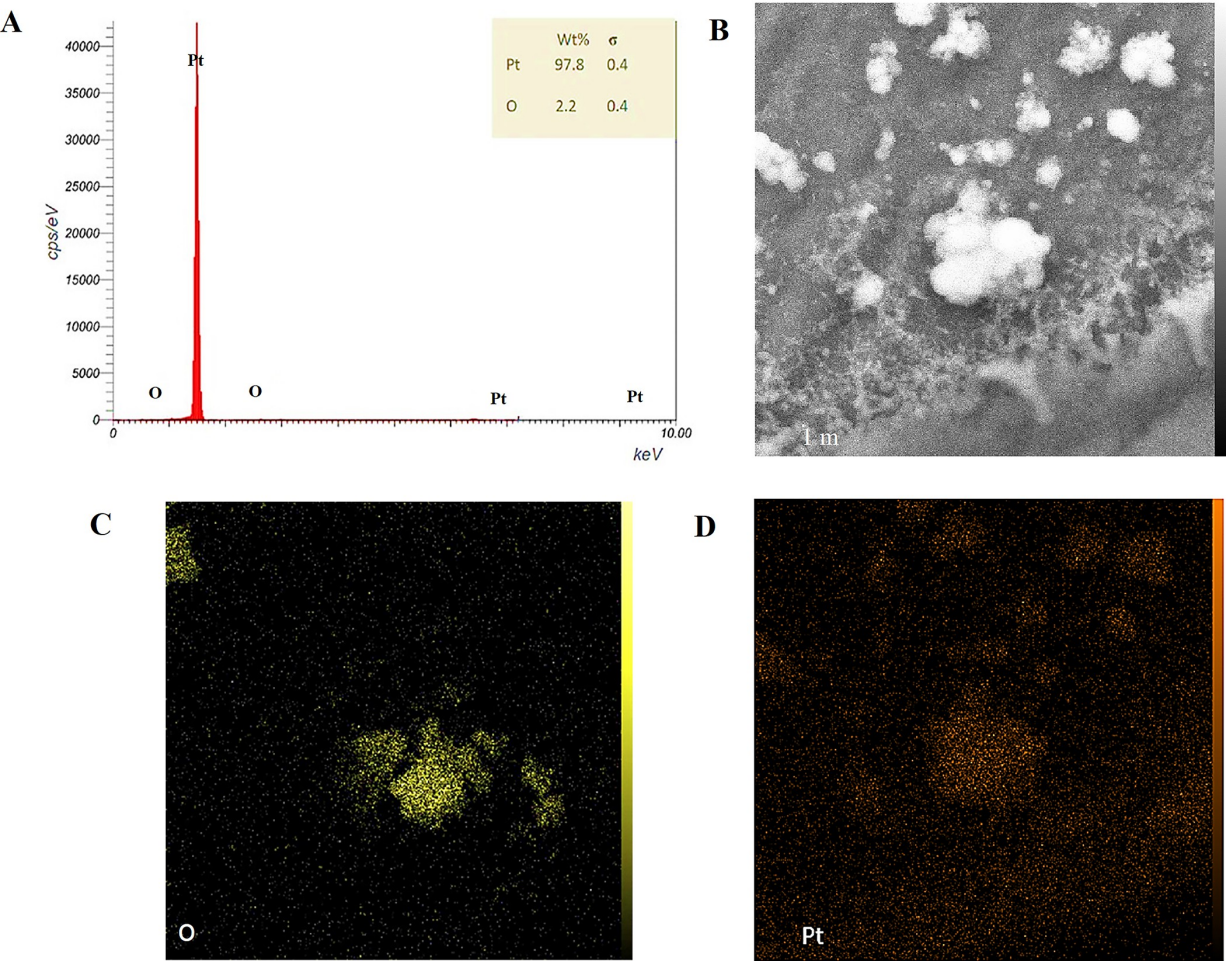
(A) EDX analysis for Pt-NPs showing EDX spot (B) and elemental mapping (C, D) of oxygen (O) and platinum (Pt), respectively.

**Fig 6:**
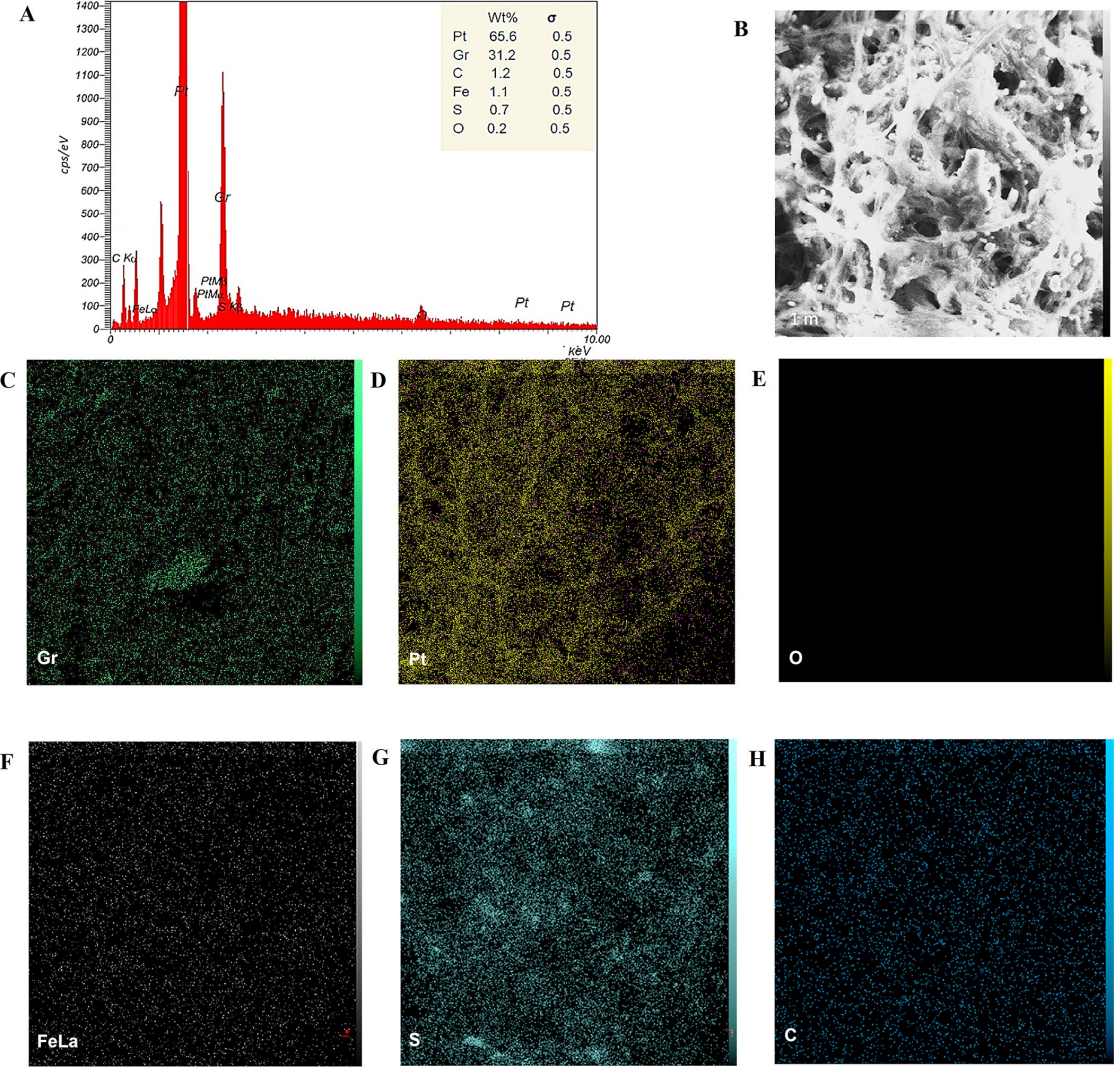
(A) EDX analysis for GRO@Pt-NPs showing EDX spot (B) and elemental mapping (C, D, E, F) of graphene (Gr), platinum (Pt), oxygen (O), iron (Fe), sulfur (S), and carbon (C), respectively.

Energy-dispersive X-ray spectroscopy (EDX) was employed to determine the elemental composition of the synthesized nanoparticles, providing a quantitative analysis of the constituent elements. For GRO-NPs, the EDX analysis revealed that the weight percentage of graphene oxide (GRO) was 90.2% of the total sample components, with a minor percentage of oxygen (O) at 9.8% (Fig 4). The presence of oxygen is attributed to the functional groups in the chemicals used in the synthesis of GRO-NPs. This high percentage of GRO indicates a successful synthesis with minimal impurities. In the case of Pt-NPs, the EDX examination showed a dominant presence of platinum (Pt) at 97.8%, with a small percentage of oxygen (2.2%) (Fig 5). The oxygen content here is likely due to the oxidation of the nanoparticle surface or residual oxygen from the synthesis process. The high Pt content in Pt-NPs confirms their purity. GRO@Pt-NPs has a different composition (Fig 6), with 65.6% platinum, 35.2% GRO, and 0.2% oxygen, confirming its core-shell structure. Small amounts of iron, sulfur, and carbon were detected [37]. The EDX investigation of GRO@Pt-NPs exposed a stable core-shell structure with a lower oxygen content than GRO-NPs and Pt-NPs. Though the EDX analysis revealed the presence of iron and sulfur elements, raising concerns about potential impurities. The EDX and mapping confirmed the uniform distribution of platinum on the graphene oxide surface.

#### 3.2.4. XRD analysis

**Fig 7:**
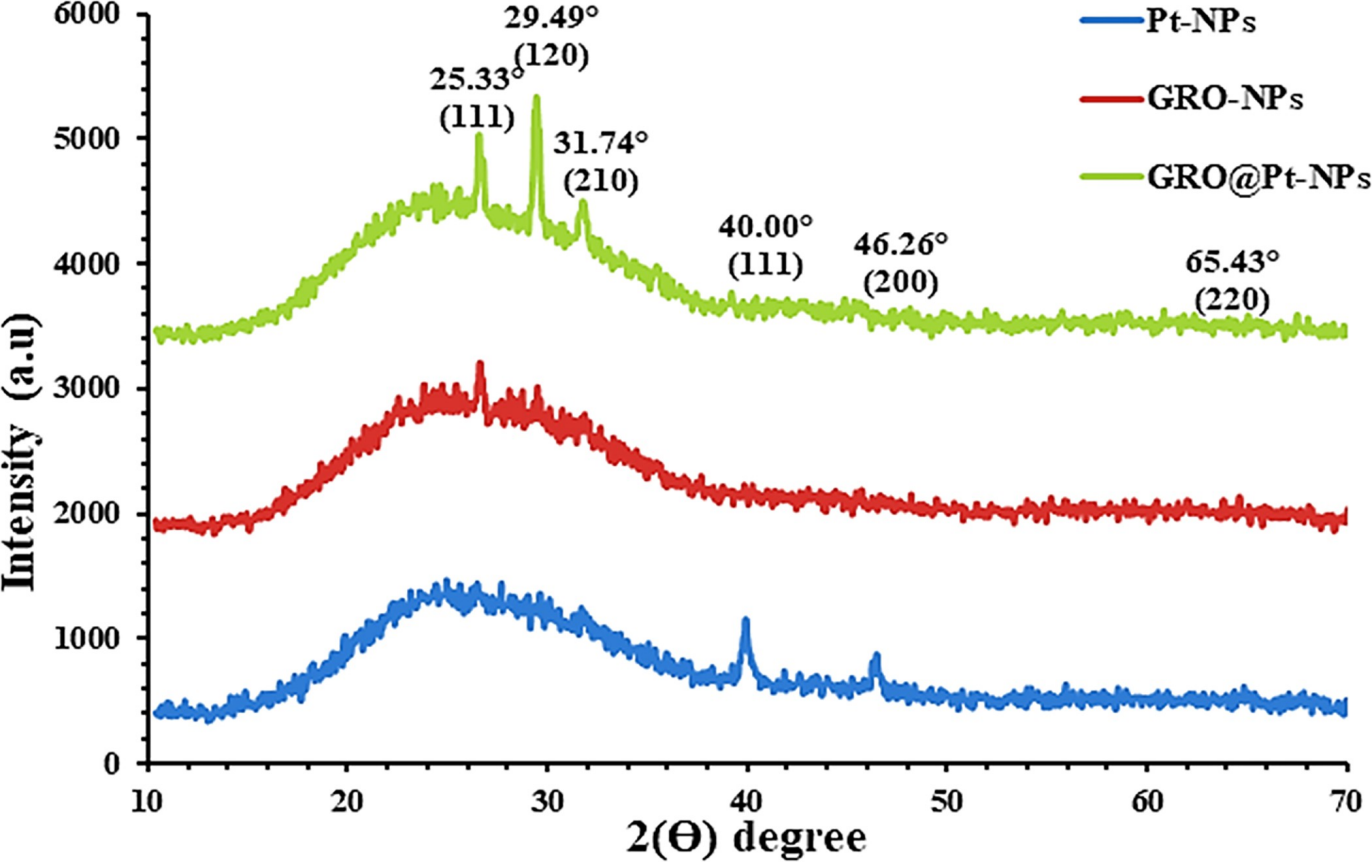
XRD analysis of Pt-NPs, GRO-NPs, and GRO@Pt-NPs.

XRD examination is an investigative method used to document crystalline materials and provides knowledge of their phase. Also, it can give information on the material’s unit cell dimensions. As seen in Fig 7, the construction and Ɵ degree of crystallinity of GRO-NPs, Pt-NPs, and GRO@Pt-NPs drop cast on a glass substrate were studied by XRD. The diffraction peak of GRO-NPs was recorded at 2θ = 25.33°, 29.49°, and 31.74°, which corresponded to the (111), (120), and (210) crystal planes, indicating that GRO-NPs has a crystalline structure consistent with the face-centered cubic (FCC) phase (JCPDS No. 00-041-1487). The presence of these specific peaks confirms the crystalline nature of the GRO-NPs. In the case of Pt-NPs, diffraction peaks were recorded at 2θ = 40.00°, 46.26°, and 66.43°, which correspond to the (111), (200), and (220) crystal planes of platinum. These peaks are characteristic of a face-centered cubic (FCC) phase (JCPDS No. 01-087-0646). The consistent frequency distribution of these peaks suggests that the Pt-NPs are uniformly crystalline.

For GRO@Pt-NPs, the XRD pattern shows diffraction peaks at 2θ = 25.33°, 29.49°, 31.74°, 40.00°, 46.26°, and 66.43°, corresponding to the (111), (120), (210), (200), and (220) crystal planes. These peaks indicate the successful formation of the composite nanoparticles. The newly appeared peaks at 40.00°, 46.26°, and 66.43° confirm the presence of platinum particles on the graphene substrate, indicating that Pt-NPs were uniformly decorated on the GRO surface. The lower peak intensity observed in the core-shell structure of GRO@Pt-NPs compared to Pt-NPs suggests a lower crystallinity, which could be due to the interaction and integration of platinum with the graphene oxide. This finding aligns with the results obtained by Feng *et al*. [36], further confirming the successful synthesis and crystalline nature of the nanoparticles. Additionally, the XRD results corroborate the TEM findings, which also indicated the successful formation of both graphene nanoparticles (GRO-NPs) and core-shell nanoparticles of graphene encapsulated by platinum (GRO@Pt-NPs), as shown in Fig 3C.

### 3.3. The antibacterial activities

**Fig 8:**
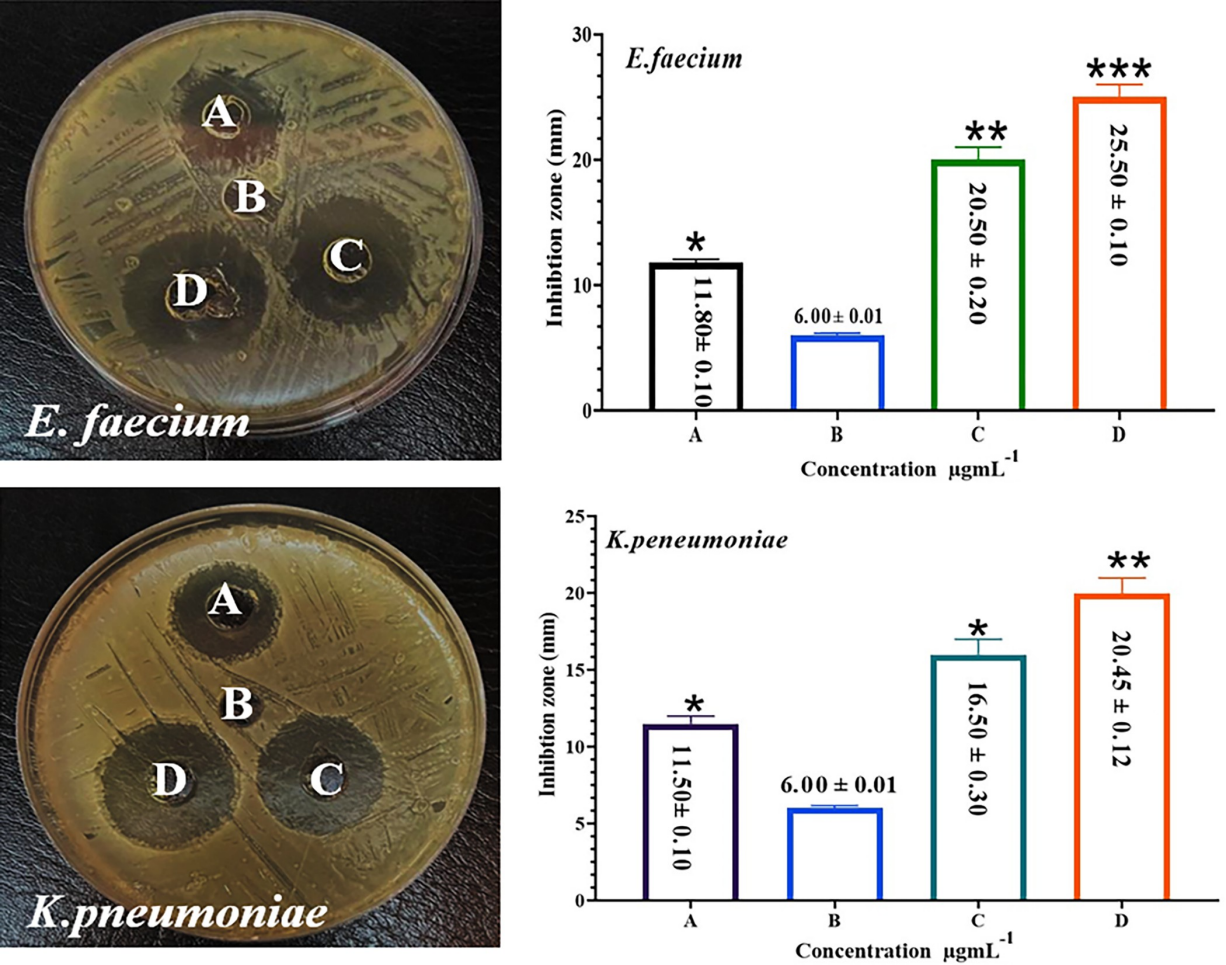
Inhibition zone of (A) GRO-NPs, (B) control negative D.W, (C) Pt-NPs, (D) GRO@Pt-NPs against *E*. *faecium* and *K*. *pneumoniae*. *p≤0.05, ** p≤0.01, *** p≤0.001.

The antibacterial actions of the GRO@Pt-NPs against two bacterial strains, *K*. *pneumoniae* and *E*. *faecium*, were tested using the agar well diffusion method. As seen in Fig 8, the treatments with 25 μgmL^-1^ of the GRO-NPs, Pt-NPs, and GRO@Pt-NPs. The highest inhibition was recorded against *E*. *faecium*. There was no inhibition at the lowest concentration against *K*. *pneumoniae* at 25 μgmL^-1^ by GRO@Pt-NPs. The observed evidence of the synergistic effects of the tested substances was the enlargement of the inhibition zones in the measured areas. In the case of *E*. *faecium*, the inhibition zones were 16 mm for GRO-NPs alone, 10.33 mm for Pt-NPs alone, and 20 mm for GRO@Pt-NPs (as shown in Fig 8). Similarly, for *K*. *pneumoniae*, the inhibition zones were 21.01 mm for GRO-NPs alone, 15.30 mm for Pt-NPs alone, and 25.05 mm for GRO@Pt-NPs (as shown in Fig 8). The latest research indicates that the prepared nanoparticles can effectively reduce bacterial growth. Among the nanoparticles studied, the synthetic GRO@Pt-NPs exhibited the largest inhibition zone against both *K*. *pneumoniae* and *E*. *faecium*. The GRO@Pt-NPs possessed significant antibacterial properties (P≤0.001) against *E*. *faecium*, which was more potent than that recorded in GRO-NPs alone or Pt-NPs alone. For *K*. *pneumoniae*, the same effect was observed, which was higher activity than that recorded in GRO-NPs alone or Pt-NPs alone (P≤0.05). The statistical significance levels shown in Fig 8 suggest that the differences in inhibition zones between the treatment groups are statistically significant. The other nanoparticles examined in the study were found to be less effective compared to GRO@Pt-NPs. Additionally, the outer membrane of the bacteria acts as a barrier, preventing the entry of negatively charged reactive oxygen species (ROS) [31,37–41].

On the other hand, Gram-positive cells have a less negative charge in their membrane, allowing negatively charged ROS to pass through [27,28,38,42]. The antimicrobial activity of GR@PTNPs is influenced by their particle size. Reducing the element size of CIP@AuNPs has been shown to improve their antibacterial action [27]. The results show that GRO@Pt-NPs display important anti-bacterial properties. Once examining two dissimilar sizes of GRO@ Pt-NPs, namely 4 >22 nm and 5–30 nm, the 22 nm GRO@Pt-NPs were found to be the most active antibacterial agent due to their small size. Moreover, earlier studies have discussed the size, shape, and toxicity mechanisms of metal oxide NPs against microbial strains [37]. These studies have exposed that nanoparticles can interact with isolated DNA molecules, leading to dose-dependent degradation and the creation of reactive oxygen species. These studies have shown that nanoparticles have the ability to interact with individual DNA molecules, resulting in the degradation of DNA in a dose-dependent manner through the generation of reactive oxygen species. GRO@Pt-NPs conjugates exhibit enhanced antibacterial effects by binding to and penetrating cell walls, thereby delivering multiple antibiotic molecules to localized areas. By treating them with antibiotics, sphere-shaped GRO@Pt-NPs with surface plasmon resonance (SPR) absorption can be easily generated. This method enables the discovery and quantification of antibiotics without the need for derivatization or expensive tools. Moreover, the conjugation of GRO-NPs with Pt-NPs eases the diffusion of drugs into microbial cells.

**Fig 9:**
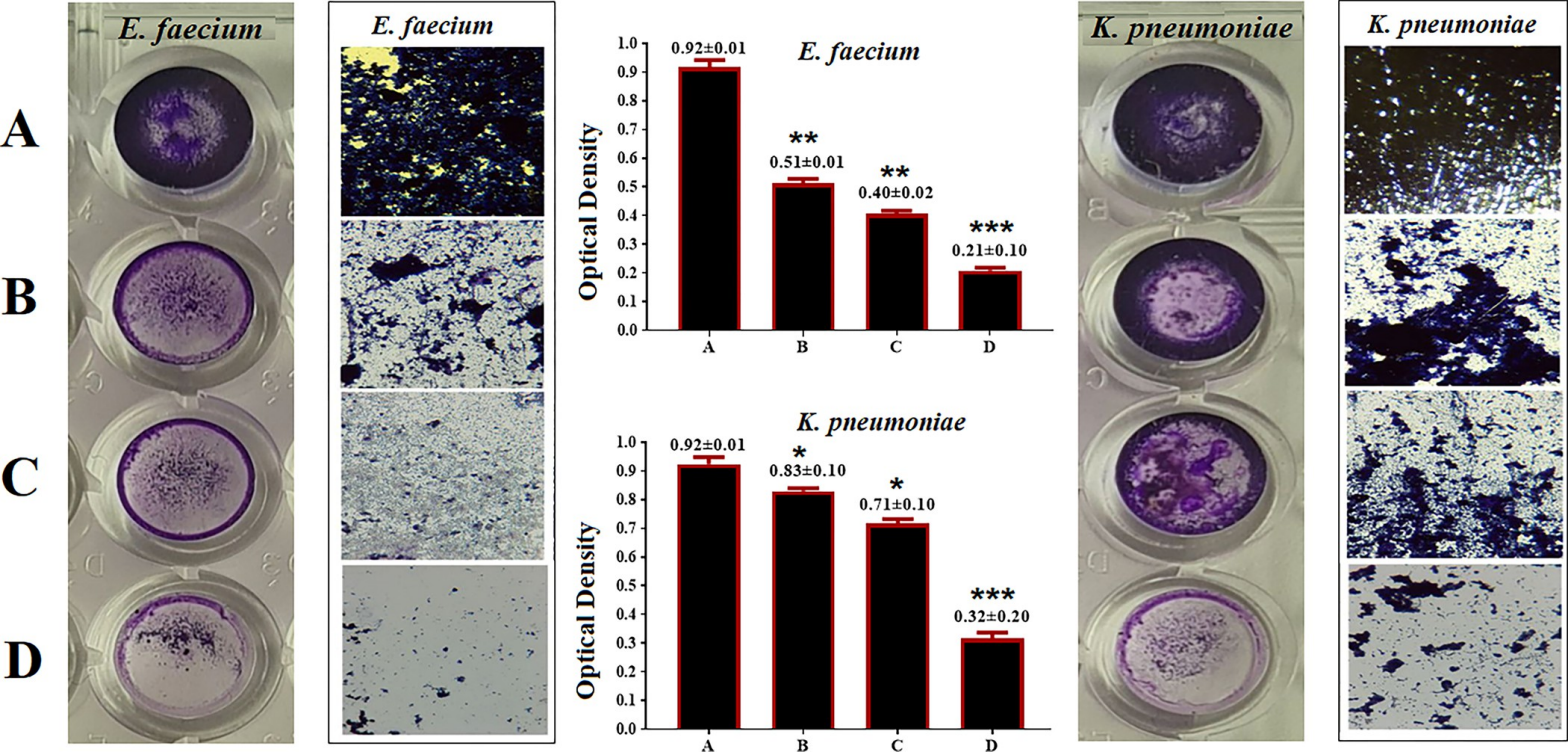
Reduces biofilm formation in *K*. *pneumoniae*, *E*. *faecium*. **(**A) Control, (B) (Pt-NPs), (C) (GRO-NPs), and (D) (GRO@Pt-NPs) stained using crystal violet. *p≤0.05, ** p≤0.01, *** p≤0.001. Magnification power is 100 X.

Fig 9 delivers a summary of the experiential outcomes of the confirmed nanotest and the anti-biofilm creation test against *E*. *faecium* and *K*. *pneumoniae*. The consequences show that all nanoparticles (Pt-NPs, GRO-NPs, and GRO@Pt-NPs) efficiently prevented the development of biofilms for together bacterial isolation, although the GRO-NPs inhibited biofilm formation with a value of 0.51. In the case of *E*. *faecium*, Pt-NPs inhibited biofilm formation with a value of 0.40. On the other hand, GRO-NPs prevented biofilm creation in *K*. *pneumoniae* with a value of 0.83. Pt-NPs also inhibited biofilm formation in *K*. *pneumoniae*, showing a value of 0.71. Additionally, the core-shell nanoparticle GRO@Pt-NPs prevented biofilm formation with a value of 0.21 and 0.32, respectively. The GRO@Pt-NPs possessed significant antibioflim activity (P≤0.001), which was higher than that observed in GRO-NPs alone or Pt-NPs alone.

Two theories have been put forth to explain the observed outcomes. Firstly, it is suggested that the presence of GRO@Pt-NPs ions hinders the development of biofilms in both bacteria. These ions are supposed to enter the microbial cells and disturb the proteins and enzymes essential for bacterial bonding. This disruption ultimately results in a decrease in biofilm formation. Additionally, the GRO@Pt-NPs impede the synthesis of exogenous polysaccharides (exopolysaccharides), which play a crucial role in biofilm formation, thereby inhibiting biofilm development. Secondly, it is proposed that the GRO@Pt-NPs enter the water channels, referred to as aquapores, that facilitate the transportation of water and nutrients across the layers of polysaccharides on the bacterial cell wall. By entering these channels, the GRO@Pt-NPs prevent the formation of biofilms [43,44].

## 4. Conclusions

In this study, we successfully synthesized a novel core-shell structure of graphene oxide-supported platinum nanoparticles (GRO@Pt-NPs) using a two-step pulsed laser ablation in liquid (PLAL) technique. The resulting nanoparticles were characterized using UV-VIS spectroscopy, transmission electron microscopy (TEM), energy dispersive X-ray (EDX), mapping tests, and X-ray diffraction (XRD), confirming the formation and structure of the GRO@Pt-NPs with an average particle size of 26 nm. Our findings demonstrated that GRO@Pt-NPs exhibit excellent antibacterial and anti-biofilm activities against *K*. *pneumoniae* and *E*. *faecium*. Indicating a synergistic enhancement of antibacterial efficacy in the core-shell configuration. This innovative synthesis technique offers a rapid and efficient way to produce nanoparticles with high clinical potential for tackling bacterial infections, especially in the context of rising multi-drug resistance. Overall, this research highlights the promising application of GRO@Pt-NPs in developing advanced antibacterial materials and opens new avenues for future investigations into their biosafety and therapeutic efficacy.

## References

[1] Mendivil Palma MI. Synthesis and characterization of metal nanoparticles by pulsed laser ablation in liquid media (PLAL). Universidad Autónoma de Nuevo León; 2015.

[2] Semaltianos NG. Nanoparticles by laser ablation. Crit Rev solid state Mater Sci. 2010;35: 105–124.

[3] Jan H, Khan MA, Usman H, Shah M, Ansir R, Faisal S, et al. The Aquilegia pubiflora (Himalayan columbine) mediated synthesis of nanoceria for diverse biomedical applications. RSC Adv. 2020;10: 19219–19231. doi: 10.1039/d0ra01971b 35515478 PMC9054089

[4] Abou El-Nour KMM, Eftaiha A, Al-Warthan A, Ammar RAA. Synthesis and applications of silver nanoparticles. Arab J Chem. 2010;3: 135–140.

[5] Kelly KL, Coronado E, Zhao LL, Schatz GC. The optical properties of metal nanoparticles: the influence of size, shape, and dielectric environment. The Journal of Physical Chemistry B. ACS Publications; 2003. pp. 668–677.

[6] Akther T, Khan MS, Srinivasan H. Novel silver nanoparticles synthesized from anthers of Couroupita guianensis Abul. control growth and biofilm formation in human pathogenic bacteria. Nano Biomed Eng. 2018;10: 250–257.

[7] MUHARRAM RA KAMADJAJA D, SALSABILLA SA, SUCIADI SP, BUDI HS. Determination of Minimum Inhibitory Concentration and Minimum Bactericidal Concentration of Silver Ion Against Staphylococcus Aureus. Int J Pharm Res. 2020;12.

[8] Imarah AA, Jabir MS, Abood AH, Sulaiman GM, Albukhaty S, Mohammed HA, et al. Graphene oxide-induced, reactive oxygen species-mediated mitochondrial dysfunctions and apoptosis: high-dose toxicity in normal cells. Nanomedicine. 2023;18: 875–887. doi: 10.2217/nnm-2023-0129 37470184

[9] Fadhil WA, Jabbar II, Ali EH, Sulaiman GM, Khan RA, Mohammed HA. Freshly Prepared Graphene Oxide Nanoparticles’ Wound-Healing Potential and Antibacterial Activity Specifically Against Staphylococcus aureus: In Vivo Efficacy and Clinical Isolate Evaluation. Plasmonics. 2024; 1–11.

[10] Hussein MO. Performance of graphene-based and polyether-ether-ketone polymers as removable partial denture esthetic clasp materials after cyclic fatigue. Polymers (Basel). 2022;14: 2987. doi: 10.3390/polym14152987 35893952 PMC9331630

[11] Kumar P, Huo P, Zhang R, Liu B. Antibacterial Properties of Graphene-Based Nanomaterials. Nanomaterials. 2019;9: 737. doi: 10.3390/nano9050737 31086043 PMC6567318

[12] Rugaie O Al, Jabir M, Kadhim R, Karsh E, Sulaiman GM, Mohammed SAA, et al. Gold Nanoparticles and Graphene Oxide Flakes Synergistic Partaking in Cytosolic Bactericidal Augmentation: Role of {ROS} and {NOX}2 Activity. Microorganisms. 2021;9: 101. doi: 10.3390/microorganisms9010101 33466290 PMC7824746

[13] Al-Omar MS, Jabir M, Karsh E, Kadhim R, Sulaiman GM, Taqi ZJ, et al. Gold Nanoparticles and Graphene Oxide Flakes Enhance Cancer Cells’ Phagocytosis through Granzyme-Perforin-Dependent Biomechanism. Nanomaterials. 2021;11: 1382. doi: 10.3390/nano11061382 34073808 PMC8225074

[14] Parnianchi F, Nazari M, Maleki J, Mohebi M. Combination of graphene and graphene oxide with metal and metal oxide nanoparticles in fabrication of electrochemical enzymatic biosensors. Int Nano Lett. 2018;8: 229–239.

[15] Georgakilas V, Tiwari JN, Kemp KC, Perman JA, Bourlinos AB, Kim KS, et al. Noncovalent functionalization of graphene and graphene oxide for energy materials, biosensing, catalytic, and biomedical applications. Chem Rev. 2016;116: 5464–5519. doi: 10.1021/acs.chemrev.5b00620 27033639

[16] Wu J-B, Lin M-L, Cong X, Liu H-N, Tan P-H. Raman spectroscopy of graphene-based materials and its applications in related devices. Chem Soc Rev. 2018;47: 1822–1873. doi: 10.1039/c6cs00915h 29368764

[17] Qiu C, Zhou H, Cao B, Sun L, Yu T. Raman spectroscopy of morphology-controlled deposition of Au on graphene. Carbon N Y. 2013;59: 487–494.

[18] Wu S, Liu J, Tian Z, Cai Y, Ye Y, Yuan Q, et al. Highly dispersed ultrafine Pt nanoparticles on reduced graphene oxide nanosheets: in situ sacrificial template synthesis and superior electrocatalytic performance for methanol oxidation. ACS Appl Mater Interfaces. 2015;7: 22935–22940. doi: 10.1021/acsami.5b06153 26435201

[19] Tavakkoli M, Holmberg N, Kronberg R, Jiang H, Sainio J, Kauppinen EI, et al. Electrochemical activation of single-walled carbon nanotubes with pseudo-atomic-scale platinum for the hydrogen evolution reaction. Acs Catal. 2017;7: 3121–3130.

[20] Mohammed HA, Amin MA, Zayed G, Hassan Y, El-Mokhtar M, Saddik MS. In vitro and in vivo synergistic wound healing and anti-methicillin-resistant Staphylococcus aureus (MRSA) evaluation of liquorice-decorated silver nanoparticles. J Antibiot (Tokyo). 2023; 1–10. doi: 10.1038/s41429-023-00603-4 36854977

[21] Jawad KH, Jamagh FK, Sulaiman GM, Hasoon BA, Albukhaty S, Mohammed HA, et al. Antibacterial and antibiofilm activities of amikacin-conjugated gold Nanoparticles: A promising formulation for contact lens preservation. Inorg Chem Commun. 2024;162: 112286.

[22] Hussein NN, Al-Azawi K, Sulaiman GM, Albukhaty S, Al-Majeed RMA, Jabir M, et al. Silver-cored Ziziphus spina-christi extract-loaded antimicrobial nanosuspension: overcoming multidrug resistance. Nanomedicine. 2023;18: 1839–1854. doi: 10.2217/nnm-2023-0185 37982771

[23] Ikram M, Haider A, Imran M, Haider J, Naz S, Ul-Hamid A, et al. Assessment of catalytic, antimicrobial and molecular docking analysis of starch-grafted polyacrylic acid doped BaO nanostructures. Int J Biol Macromol. 2023;230: 123190. doi: 10.1016/j.ijbiomac.2023.123190 36623614

[24] Muteeb G. Nanotechnology—A Light of Hope for Combating Antibiotic Resistance. Microorganisms. 2023;11: 1489. doi: 10.3390/microorganisms11061489 37374990 PMC10302692

[25] Akram F, Imtiaz M, ul Haq I. Emergent crisis of antibiotic resistance: A silent pandemic threat to 21st century. Microb Pathog. 2023;174: 105923. doi: 10.1016/j.micpath.2022.105923 36526035

[26] Pastukhov AI, Belyaev IB, Bulmahn JC, Zelepukin I V, Popov AA, Zavestovskaya IN, et al. Laser-ablative aqueous synthesis and characterization of elemental boron nanoparticles for biomedical applications. Sci Rep. 2022;12: 9129. doi: 10.1038/s41598-022-13066-8 35650237 PMC9159993

[27] Hasoon BA, Jawad KH, Abdulsahib SS. Synthesis of Ciprofloxacin-Conjugated Gold Nanoparticles and their Study Antibacterial Effects on Growth Biofilm Formation Through Nebulizer Mask Against Respiratory Infection. Plasmonics. 2023; 1–15.

[28] Mohammed AA, Jawad KH, Çevik S, Sulaiman GM, Albukhaty S, Sasikumar P. Investigating the antimicrobial, antioxidant, and anticancer effects of elettaria cardamomum seed extract conjugated to green synthesized silver nanoparticles by laser ablation. Plasmonics. 2023; 1–14.

[29] Hasan DMA, Hasoon BA, Abdulwahab AI, Jawad KH. Biological activities of Ethanolic Extract Produced by Cucurbita pepo plant. Revis Bionatura 2022; 7 (2) 19. s Note: Bionatura stays neutral with regard to jurisdictional claims in …; 2022.

[30] Al-Saadi HK, Awad HA, Saltan ZS, Hasoon BA, Abdulwahab AI, Al-azawi KF, et al. Antioxidant and Antibacterial Activities of Allium sativum Ethanol Extract and Silver Nanoparticles. Trop J Nat Prod Res. 2023;7.

[31] Visser D. Atomic absorption spectroscopy, principles and applications. Technol Networks Anal Sep Available https://www/Technolcom/analysis/articles/atomic-absorption-spectroscopy-principles-and-applications-356829 (accessed 28 December 2022). 2021.

[32] Sharma M, Easha P, Tapasvi G, Reetika R. Nanomaterials in biomedical diagnosis. Nanomaterials in Diagnostic Tools and Devices. Elsevier; 2020. pp. 57–83.

[33] Gragossian A, Tavassoli SH, Shokri B. Laser ablation of aluminum from normal evaporation to phase explosion. J Appl Phys. 2009;105.

[34] Ghadiri Zahrani E, Alexopoulou VE, Papazoglou EL, Azarhoushang B, Markopoulos A. An Experimental and Numerical Study of the Laser Ablation of Bronze. Machines. 2024;12: 63.

[35] Liu S, Zhang N, Xu Y. Core–shell structured nanocomposites for photocatalytic selective organic transformations. Part Part Syst Charact. 2014;31: 540–556.

[36] Tanaka Y. Synthesis of nanosize particles in thermal plasmas. Handb Therm Sci Eng Kulacki F, Ed; Springer Cham, Ger. 2018; 2791–2828.

[37] Jabir MS, Nayef UM, Kadhim WKA. Polyethylene glycol-functionalized magnetic (Fe3O4) nanoparticles: A novel DNA-mediated antibacterial agent. Nano Biomed Eng. 2019;11: 18–27.

[38] Jawad KH. Laser ablation mediated ZnO nanoparticles inhibit growth and biofilm forming potential of urinary tract bacterium Proteus mirabilis. Adv Nat Sci Nanosci Nanotechnol. 2023;14: 15002.

[39] Ahire JJ, Neveling DP, Hattingh M, Dicks LMT. Ciprofloxacin-eluting nanofibers inhibits biofilm formation by Pseudomonas aeruginosa and a methicillin-resistant Staphylococcus aureus. PLoS One. 2015;10: e0123648. doi: 10.1371/journal.pone.0123648 25853255 PMC4390291

[40] Maeh RK, Jaaffar AI, Al-Azawi KF. Preparation of Juniperus extract and detection of its antimicrobial and antioxidant activity. Iraqi J Agric Sci. 2019;50.

[41] Mohammed WH, Hussein AA, Saleem MMNM. Influence of antibiotic and stick sweet cherry (Prunus aviam) on pathogenic bacteria and evaluation of Tissues Bioavailability, Bioactive phytochemical compounds & Functional properties. Plant Arch. 2020;20: 298–308.

[42] Mahdi LH, Hasoon BA, Sulaiman GM, Mohammed HA, Jawad KH, Al-Dulimi AG, et al. Anti-microbial efficacy of l-glutaminase (EC 3.5. 1.2) against multidrug-resistant Pseudomonas aeruginosa infection. J Antibiot (Tokyo). 2024;77: 111–119. doi: 10.1038/s41429-023-00678-z 38017084

[43] Hasoon BA, Jawad KH, Mohammed IS, Hussein NN, Al-azawi KF, Jabir MS. Silver nanoparticles conjugated amoxicillin: A promising nano-suspension for overcoming multidrug resistance bacteria and preservation of endotracheal tube. Inorg Chem Commun. 2024; 112456.

[44] Yosif HM, Hasoon BA, Jabir MS. Laser Ablation for Synthesis of Hydroxyapatite and Au NP Conjugated Cefuroxime: Evaluation of Their Effects on the Biofilm Formation of Multidrug Resistance Klebsiella pneumoniae. Plasmonics. 2023; 1–15.

